# A comparative perception and impact of pictorial warnings on cigarette packaging among Malaysian smokers and non-smokers

**DOI:** 10.7717/peerj.18713

**Published:** 2024-12-18

**Authors:** Nurul Asyikin Yahya, Nur Aqilah Mohamed Kutty, Nurul Afira Saleh

**Affiliations:** 1 Department of Family Oral Health, Faculty of Dentistry, Universiti Kebangsaan Malaysia, Kuala Lumpur, Wilayah Persekutuan Kuala Lumpur, Malaysia; 2 Faculty of Dentistry, Universiti Kebangsaan Malaysia, Kuala Lumpur, Wilayah Persekutuan Kuala Lumpur, Malaysia

**Keywords:** Cigarette packaging, Impact, Perception, Smokers, non-smokers, Pictorial warning, Malaysian

## Abstract

**Introduction:**

In 2003, the World Health Organization (WHO) recommended that pictorial warnings on tobacco products be used to educate consumers on the negative health impacts associated with tobacco use. This study aimed to assess the effectiveness of pictorial warnings on cigarette packaging among smokers and non-smokers.

**Methods:**

A cross-sectional study using a self-administered questionnaire was conducted in Kuala Lumpur. Four components assessed the respondents’ socio-demographics, smoking status, perceptions, and impact of pictorial warnings. Six standard cigarette pictorial warning images were included in the questionnaire. Data collected were analysed using SPSS version 29.0. Frequencies and percentages were used for categorical data, while means and standard deviations were used for continuous data. Statistically significant results were set at *p*-value < 0.05. Non-parametric tests, the Chi-square test and the One-Way ANOVA test were used to calculate the differences between the variables, as the data was not normally distributed.

**Results:**

Three hundred seventy-eight respondents participated in this study, with smokers and non-smokers divided equally, 189. Most respondents were 20–29 years old (*n* = 223, 59.0%) and had tertiary education (*n* = 207, 54.8%). Most smokers were male (*n* = 172, 91.0%), and most non-smokers were female (*n* = 119, 63.0%). Most respondents (*n* = 364, 96.3%, *p* = 0.276) noticed the pictorial warnings on cigarette packs. Nevertheless, significantly (*p* < 0.001) more smokers (*n* = 73, 38.6%) seldomly read the content compared to non-smokers (*n* = 57, 30.2%). Most smokers (*n* = 48, 12.7%) sometimes consider quitting smoking upon exposure to pictorial warnings on cigarette packs. However, most non-smokers (*n* = 161, 42.6%) never had the urge to smoke upon looking at the pictorial warnings. The most impactful image on cigarette packs among smokers was ‘Lung cancer’ (*n* = 74, 39.2%), while for non-smokers was ‘Mouth cancer’ (*n* = 59, 31.2%) with *p* < 0.001.

**Conclusion:**

While non-smokers demonstrated greater engagement with and emotional responses to the warnings, smokers showed less frequent interaction and a tendency toward desensitisation. Although pictorial warnings play a vital role in raising awareness of the health risks of smoking, particularly lung and mouth cancer, their effectiveness in encouraging smoking cessation among smokers remains limited.

## Introduction

In 2019, smoking claimed the lives of 7.69 million individuals globally (GBD 2019 Tobacco Collaborators, 2021), resulting in one death every 4 seconds due to smoking-related illnesses. Consequently, tobacco smoking stands out as one of the leading causes of mortality worldwide. It is associated with various health complications, including lung cancer, heart disease, stroke, and chronic obstructive pulmonary disease (COPD) (World Health Organization (WHO), 2023). In Malaysia alone, smoking is responsible for approximately 20,000 deaths annually (Lim et al., 2022). Conversely, cigarette smoking has declined, dropping from 33.3% in 2000 to a targeted prevalence of 20.9% in 2025 (World Health Organization (WHO), 2013). This decline is likely attributable to a range of tobacco prevention initiatives, efforts to promote smoking cessation, and heightened public awareness regarding the adverse effects of smoking and exposure to secondhand smoke (Levy, Li & Yuan, 2019). Promoting smoking cessation is among the strategies aimed at assisting smokers in quitting or reducing their cigarette consumption by increasing the frequency of smoking cessation attempts to reduce the morbidity and mortality associated with cigarette use (World Health Organization (WHO), 2013; Levy, Li & Yuan, 2019; Hughes, 2003).

Pictorial health warnings on tobacco products have proven effective in informing consumers about the harmful effects of tobacco use, as stipulated in Article 11 of the Framework Convention on Tobacco Control (FCTC) by the World Health Organization (WHO) (2021). Compared to text-only health warnings, pictorial warnings on cigarette packages are more likely to capture attention, educate individuals about health risks, elicit more robust emotional responses, and motivate smokers to quit or reduce their tobacco consumption (Cornacchione Ross et al., 2023). Specific strategies outlined in the guidelines include placing pictorial warnings on the front and back of the packaging in undamaged areas upon opening, ensuring the display size covers 30 to 50 per cent or more of the main display area with prominently bold health messages, utilising culturally appropriate images, employing contrasting colours, and rotating pictorials. These guidelines are designed to enhance visibility and optimise the effectiveness of conveyed messages (World Health Organization (WHO), 2021).

Countries such as Brazil, Thailand, Singapore, Hong Kong, Chile, Australia and Canada that adopted pictorial warnings experienced a reduction in cigarette consumption due to increased exposure to these warnings and enhanced health awareness (Rekha & Anjum, 2012). In Malaysia, the introduction of pictorial warnings was facilitated by enacting the Control of Tobacco Product (Amendment) Regulations (CTPR) in 2008. Effective January 1, 2009, cigarette packs sold in Malaysia became compulsory to feature six rotating pictorial warnings covering 40% of the front and 60% of the back of the main areas of each pack (Ministry of health Malaysia, 2008).

A specific study conducted a comprehensive review of available literature concerning the perceived impact of pictorial health warnings on smoking behaviour changes across various Asian countries, encompassing Malaysia, China, Pakistan, Jordan, Thailand, India, Bangladesh, Turkey, Qatar, Laos, and Indonesia (Ratih & Susanna, 2018). They concluded that pictorial health warnings are perceived as more effective in preventing smoking initiation among non-smokers and in encouraging smoking cessation among smokers (Ratih & Susanna, 2018). While local researchers have investigated the efficacy and effectiveness of pictorial warnings in enhancing tobacco awareness among adult smokers in Malaysia, particularly regarding their intent to quit (Institute of Public Health (IPH), 2012a), there is still a lack of research exploring the comparison between smokers and non-smokers to gain deeper insights, as well as examining the long-term effects of pictorial warnings on smoking behaviour in the Malaysian context.

Pictorial warnings represent a distinctive aspect of tobacco control initiatives as they are encountered directly during smoking. This means nearly every smoker is exposed to these warnings, with pack-a-day smokers encountering them over 7,000 times annually (Hammond et al., 2003). Due to this extensive reach and frequency of exposure, even if the impact of pictorial warnings on individual smokers is modest, they hold the potential to exert a substantial influence on smoking behaviour at the population level (Hammond et al., 2003).

The ongoing discussion regarding the enduring impact of pictorial warnings on cigarette packaging in reducing smoking rates prompts further investigation. Thus, the primary objective is to examine the perception and effects of pictorial warnings on smokers and non-smokers, including their smoking behaviour. The secondary objective involves comparing the perceptions of smokers and non-smokers regarding pictorial warnings on cigarette packaging and assessing the effectiveness of specific images used on pictorial warnings.

## Materials and Methods

### Study design

A cross-sectional study was conducted in Kuala Lumpur using a self-administered questionnaire. Ethical approval was obtained from the UKM Research Ethics Committee (UKMREC) with reference no: UKM PPI/111/8/JEP-2023-427. A purposive sampling method was used for this study. Respondents were invited to join the study period from October 2023 till January 2024. The sample size was calculated using the Raosoft Sample Size Calculator, and the recommended sample size was 378 for a 95% confidence level, at a 5% margin of error, for an unknown population. The study divided respondents into two groups according to their tobacco usage status. Thus, the sample size for smokers and non-smokers was divided equally into 189, respectively. Participants who fulfilled the inclusion criteria were aged 15 years and above and could understand Malay and English were invited to partake. Exclusion criteria included non-illiterate people. Written consent was obtained from all participants at the time of recruitment.

A self-administered questionnaire consisting of four components in both English and Malay was used as a data collection instrument. The questions were adapted from other studies (Rekha & Anjum, 2012; Hammond et al., 2003; Ratneswaran et al., 2016). The four components are the socio-demographic details, tobacco use assessment, perception and knowledge of pictorial warnings, and impact of pictorial warnings on participants. The rating scales used in this survey were a 5-point- and 10-point Likert scales.

**Figure 1:**
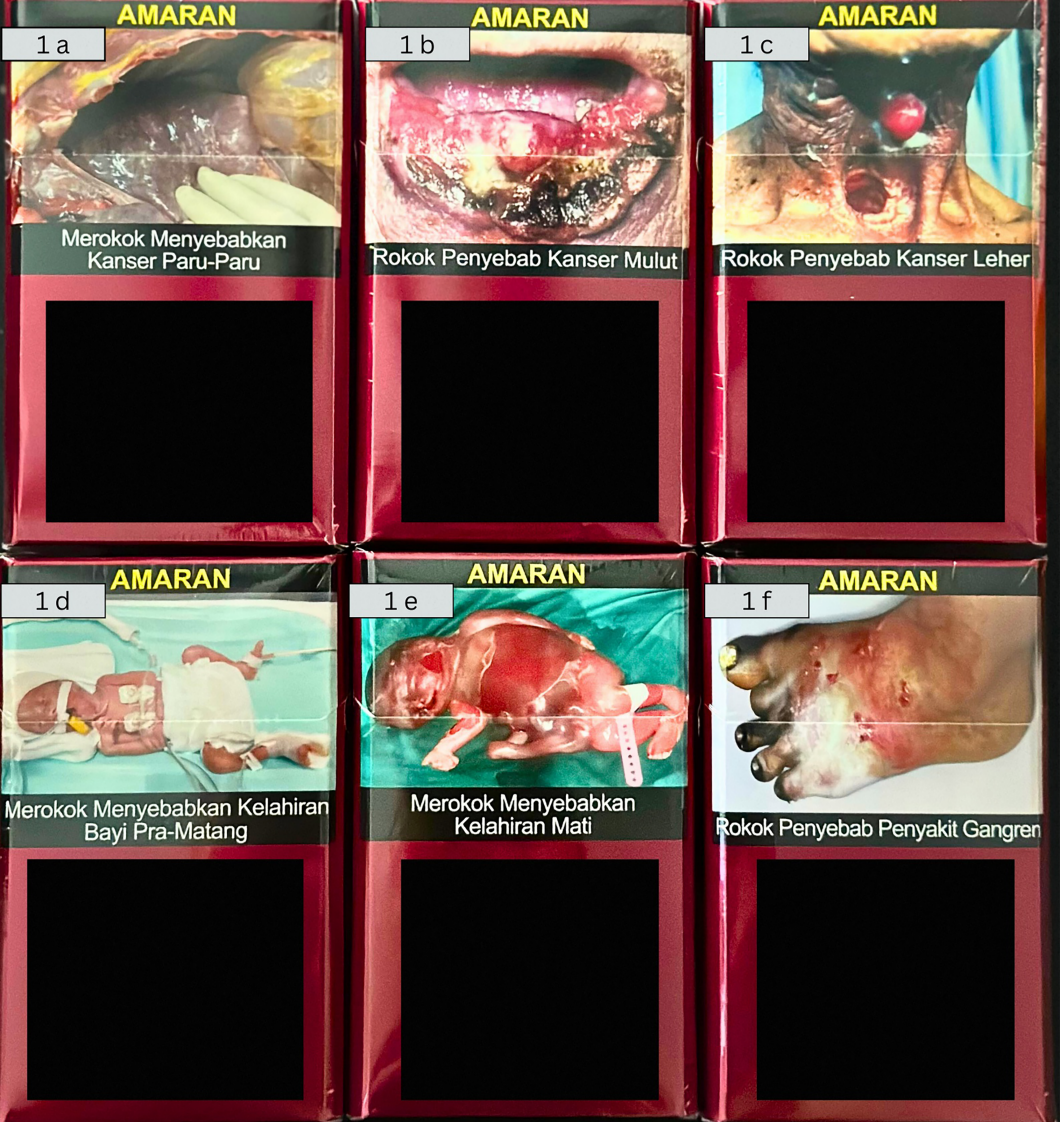
Pictorial warnings on cigarette packaging. From upper left: (A) Premature birth; (B) Lung cancer; (C) Miscarriage. From lower left: (D) Mouth cancer; (E) Gangrene; (F) Neck cancer.

The questionnaire included six standard cigarette pictorial warning images (Fig. 1). The images were mouth cancer, neck cancer, gangrene, lung cancer, miscarriage, and premature birth. Participants were shown the six pictorial warnings (Fig. 1). The participants’ emotional reaction to the pictures was observed by asking them to rate their feelings of “fear,” “disgust,” or “avoiding looking at the pictures” using a Likert scale of 1 to 10 after viewing the images.

### Data analysis

The statistical analysis of the data collected was analysed using the Statistical Package for the Social Science (SPSS) version 29.0 (IBM, Armonk, New York). Frequencies and percentages were calculated for categorical data. Means and standard deviation were used for continuous data. Non-parametric tests, the Chi-square test and the One-Way ANOVA test were used to calculate the differences between the variables, as the data was not normally distributed. Statistically significant differences were set at *p*-value < 0.05.

A Dental Public Health specialist assessed the questionnaire’s content validity. Then, a pretest was carried out with a sample of 20 individuals who were not part of the study to assess the questionnaire’s face validity and reliability. Based on the feedback and suggestions for improvement that arose during the pretest phase, final amendments and modifications were made.

## Results

### Demographic characteristics of respondents

Three hundred and seventy-eight respondents participated in this study. The demographic data for the participants are summarised in Table 1. Most respondents (*n* = 223, 58.9%) were between the ages of 20–29. More smokers were male (*n* = 172, 91%) than non-smokers, who were primarily female (*n* = 119, 63%). Most respondents (*n* = 207, 54.7%) had tertiary education. There is a statistically significant difference (*p*-value < 0.001) found between respondents’ smoking status, gender, and education level.

**Table 1:** Sociodemographic characteristics of the respondents (*n* = 378).

	Smoker *n* = 189		Non-smoker *n* = 189		*p*-value*
Demographics	*n*	%	*n*	%	
Age group					<0.001
15–20	5	2.6	20	10.6	
20–29	103	54.5	120	63.5	
30–39	46	24.3	15	7.9	
40–49	16	8.5	8	4.2	
50–59	9	4.8	11	5.8	
60 and above	10	5.3	15	7.9	
Sex					<0.001
Male	172	91	70	37	
Female	17	9	119	63	
Education level					<0.001
Primary school	6	3.2	0	0	
Secondary school	69	36.5	13	6.9	
Pre-university/STPM/Diploma/Degree	71	37.6	136	72	

**Note:**

* Statistically significant difference at *p* < 0.05.

#### Tobacco use assessment of smokers

Table 2 describes the tobacco usage of smokers. One hundred and eighty-nine participants who were smokers were assessed for their tobacco use. A majority of the smokers (*n* = 105, 27.8%) reported smoking every day, followed by 60 (15.9%) who seldom smoked. Only 24 (6.3%) reported smoking at least once a week. Most smokers (*n* = 81, 42.9%) reported using tobacco for less than 5 years, least smokers (*n* = 40, 10.6%) smoked more than 10 years. Another 40 (18%) smoked between 5 to 10 years. Most of the smokers (*n* = 109, 28.8%) reported smoking less than ten cigarettes per day, and only 13 (3.4%) smoked more than 20 cigarettes per day.

**Table 2:** Tobacco use assessment of smokers (*n* = 189).

Tobacco use assessment	Smokers (*n* = 189)	
	*n*	%
Frequency of tobacco use		
Everyday	105	55.6
At least once a week	24	12.7
Seldom	60	31.7
Duration of tobacco use		
Less than 5 years	81	42.9
5–10 years	40	21.2
More than 10 years	68	35.9
Number of cigarettes		
<10	109	57.7
10–20	67	35.4
>20	13	6.9

#### Perception towards pictorial warnings among respondents

Table 3 shows the respondents’ perception towards pictorial warnings on cigarette packaging. Most respondents (*n* = 374, 96.3%) noticed the pictorial warnings on cigarette packaging. Hence, statistically significant differences were not found between smokers and non-smokers (*p*-value = 0.276). A statistically significant difference was found between smokers and non-smokers in the frequency of reading pictorial warnings (*p*-value < 0.001). Most smokers (*n* = 73, 38.6%) rarely read pictorial warnings compared to non-smokers (*n* = 57, 30.2%). Most non-smokers (*n* = 69, 36.5%) sometimes read pictorial warnings compared to smokers (*n* = 44, 23.3%). Most smokers (*n* = 61, 32.3%) think about pictorial warnings compared to non-smokers (*n* = 51. 27.0%). While most non-smokers (*n* = 56, 29.6%) sometimes think about the pictorial warnings. Next, most smokers (*n* = 82, 43.4%) never discussed the pictorial warnings with their peers as opposed to non-smokers (*n* = 41, 21.7%). Meanwhile, most non-smokers (*n* = 59, 31.2%) sometimes discussed with peers compared to smokers (*n* = 42, 22.2%). A statistically significant difference was noted in the frequency of discussing pictorial warnings between smokers and non-smokers (*p*-value < 0.001). Lastly, most smokers (*n* = 71, 37.6%) never thought about pictorial warnings when they were not in sight compared to non-smokers (*n* = 37, 19.6%). Meanwhile, most non-smokers (*n* = 56.6%) sometimes thought about the pictorial warnings when not in sight as opposed to smokers (*n* = 43, 22.8%). Similarly, a statistically significant difference was found in the frequency of thinking about pictorial warnings when not in sight between smokers and non-smokers (*p*-value < 0.001).

**Table 3:** The perception of the respondents towards pictorial warnings on cigarette packaging.

Perception of respondents	Smoker *n* = 189		Non-smokers *n* = 189		*p*-value*
	*n*	%	*n*	%	
Notice the pictorial warnings					0.276
Yes	180	95.2	184	97.4	
No	9	4.8	5	2.6	
Frequency of reading the pictorial warnings					<0.001
Never	26	13.8	6	3.2	
Rarely	73	38.6	57	30.2	
Sometimes	44	23.3	69	36.5	
Frequent	17	9.0	27	14.3	
All the time	29	15.3	30	15.9	
Frequency of thinking about pictorial warnings					<0.001
Never	45	23.8	17	9.0	
Rarely	61	32.3	51	27.0	
Sometimes	51	27.0	56	29.6	
Frequent	15	7.9	37	19.6	
All the time	17	9.0	28	14.8	
Frequency of discussing with peers					<0.001
Never	82	43.4	41	21.7	
Rarely	35	18.5	40	21.2	
Sometimes	42	22.2	59	31.2	
Frequent	17	9.0	35	18.5	
All the time	13	6.9	14	7.4	
Thinking about pictorial warnings when not in sight					<0.001
Never	71	37.6	37	19.6	
Rarely	45	23.8	43	22.8	
Sometimes	43	22.8	56	29.6	
Frequent	19	10.1	29	15.3	
All the time	11	5.8	24	12.7	

**Note:**

* Statistically significant difference at *p* < 0.05.

#### Known health effects of tobacco use among respondents

**Figure 2:**
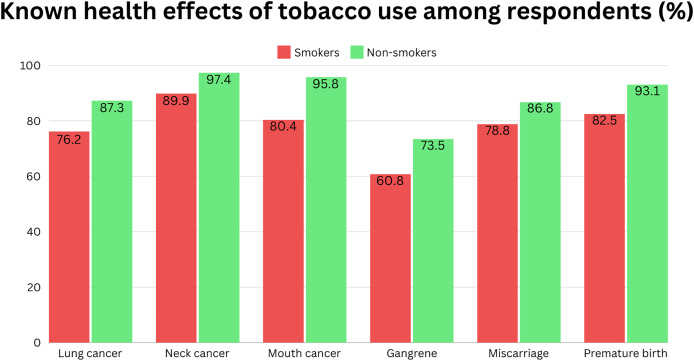
Percentage of known health effects of tobacco use among respondents (*n* = 378).

Figure 2 describes the known health effects of tobacco use among respondents. The two most recognised health effects of smoking by both groups of respondents are lung cancer (*p*-value = 0.011) and mouth cancer (*p*-value < 0.001), with 170 (89.9%) smokers and 184 (97.4%) non-smokers who answered ‘Yes’ for lung cancer while 152 (80.4%) smokers and 181 (95.8%) non-smokers answered ‘Yes’ for mouth cancer. Following these were premature birth (*p*-value < 0.001), with 156 (82.5%) smokers and 176 (93.1%), miscarriages (*p*-value = 0.048), with 149 (78.8%) smokers and 164 (86.8%) non-smokers, neck cancer (*p*-value < 0.001) with 144 (76.2%) smokers and 165 (87.3%) non-smokers and lastly gangrene (*p*-value < 0.001) with 115 (60.8%) smokers and 139 (73.5%) who answered ‘Yes’ for respective health effects.

#### Emotional impact of pictorial warnings by respondents

The emotional impact of pictorial warnings, which include fear, disgust, and avoiding looking into the image for smokers and non-smokers, were assessed respectively (Table 4). A statistically significant difference (*p*-value < 0.001) can be seen between smokers’ and non-smokers’ emotional responses to ‘fear’, ‘disgust’, and ‘avoiding looking into the pictorial images of cigarette packaging’. Non-smokers (7.61 ± 2.170) showed a higher fear level than smokers (4.08 ± 2.621). Alongside the other emotional impact, non-smokers (7.75 ± 2.219) felt more disgusted towards the sight of the pictorial images compared to smokers (4.14 ± 2.774). Non-smokers (6.31 ± 2.762) tend to avoid looking into the pictorial images higher than smokers (4.04 ± 2.904).

**Table 4:** Emotional Impact of pictorial warnings by respondents (*n* = 378).

Impact	Smoker *n* = 189		Non-smoker *n* = 189		*p-*value*
	Mean	Std. Deviation	Mean	Std. Deviation	
Fear	4.08	2.621	7.61	2.170	<0.001
Disgust	4.14	2.774	7.75	2.219	<0.001
Avoid looking into the image	4.04	2.904	6.31	2.762	<0.001

**Note:**

* Statistically significant difference at *p* < 0.05.

#### Impact of pictorial warnings on quitting smoking by smokers

One hundred and eighty-nine smokers were assessed on the impact of pictorial warnings on quitting smoking (Table 5). Table 5 shows that smokers sometimes (*n* = 48, 25.4%) and rarely (*n* = 47, 24.9%) considered quitting smoking upon the sight of pictorial warnings on cigarette packs. Most (*n* = 53, 28.0%) frequently consider quitting smoking upon looking at the pictorial warning. Smokers sometimes (*n* = 53, 28.0%) consider reducing the use of cigarettes, while 40 smokers never and rarely (21.2%) consider decreasing tobacco use. Most smokers (*n* = 56, 29.6%) frequently consider reducing cigarette use upon the sight of pictorial warnings. The target group sometimes (*n* = 64, 33.9%) had thoughts about health risks regarding tobacco use upon seeing the pictorial warnings. In contrast, some smokers (*n* = 24, 12.7%) never considered the risk to health concerning the impact of pictorial warnings. Smokers mostly admit that the pictorial warnings never (*n* = 77, 40.7%) refrained themselves from smoking. Only 16.9% (*n* = 32) of smokers agree that the pictorial warning frequently refrains them from smoking.

**Table 5:** Impact of pictorial warnings on quitting smoking by smokers (*n* = 189).

Impact	Smoker *n* = 189	
	*n*	%
1. Consider to quit smoking		
Never	41	21.7
Rarely	47	24.9
Sometimes	48	25.4
Frequent	53	28.0
2. Consider to reduce use of cigarette		
Never	40	21.2
Rarely	40	21.2
Sometimes	53	28.0
Frequent	56	29.6
3. Thought about risk of health		
Never	24	12.7
Rarely	31	16.4
Sometimes	64	33.9
Frequent	70	37.0
4. Refrain from smoking		
Never	77	40.7
Rarely	36	19.0
Sometimes	44	23.3
Frequent	32	16.9

#### Impact of pictorial warnings of the initiation to smoking by non-smokers

**Figure 3:**
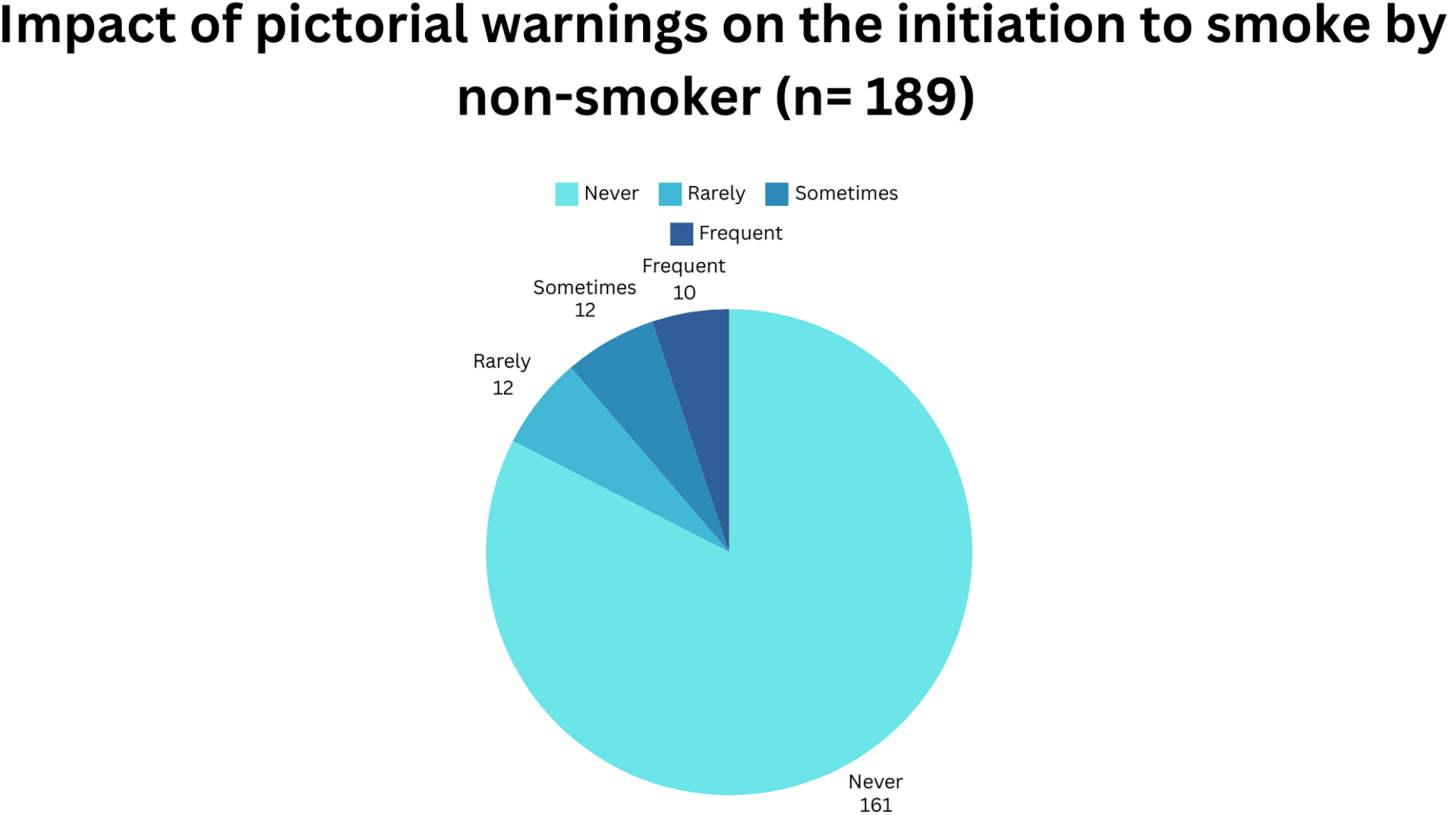
Impact of pictorial warnings on the initiation to smoking by non-smokers (*n* = 189).

Figure 3 shows the impact of pictorial warnings on the initiation of smoking by non-smokers. Most non-smokers (*n* = 161, 85.2%) never had the urge to smoke upon the sight of pictorial warnings on the cigarette packaging. It follows that 12 out of 189 rarely and sometimes (6.3%) had the urge to smoke, respectively. However, a small number of respondents frequently (*n* = 4, 2.1%) had the urge to smoke about the impact of pictorial warnings.

#### Impactful images on cigarette packs among respondents

Table 6 describes the most impactful image on cigarette packs by respondents. There is a statistically significant difference (*p*-value < 0.05) between smokers and non-smokers on impactful images on cigarette packs among respondents. The majority of smokers chose lung cancer (*n* = 74, 39.2%) as the most impactful image on cigarette packs, followed by premature birth (*n* = 41, 21.7%), neck cancer (*n* = 39, 20.6%), miscarriage (*n* = 10, 5.3%) and lastly gangrene (*n* = 6, 3.2%). However, non-smokers picked mouth cancer (*n* = 59, 31.2%) as the most impactful pictorial warnings on cigarette packs, subsequently lung cancer (*n* = 58, 30.7%), neck cancer (*n* = 45, 23.8%), premature birth (*n* = 15, 7.9%), and lastly gangrene (*n* = 5, 2.6%).

**Table 6:** Impactful images on cigarette packs among respondents (*n* = 378).

Images	Smoker *n* = 189		Non-smoker *n* = 189		*p*-value*
	*n*	%	*n*	%	
Lung cancer	74	39.2	58	30.7	<0.001
Neck cancer	39	20.6	45	23.8	
Mouth cancer	19	10.1	59	31.2	
Premature birth	41	21.7	15	7.9	
Miscarriage	10	5.3	7	3.7	
Gangrene	6	3.2	5	2.6	

**Note:**

* Statistically significant difference at *p* < 0.05.

## Discussion

In this study, the majority of participants were young adults, revealing a notable sex disparity between smokers and non-smokers, with males predominantly represented among smokers and females among non-smokers. These trends mirrored those observed in national data from the Malaysia Global Adult Tobacco Survey (GATS), 2011 (Institute of Public Health (IPH), 2012b). Remarkably, both smokers and non-smokers exhibited a propensity toward tertiary education, a contrast from the conventional association between lower educational attainment and smoking in Malaysia, as highlighted in the National Health and Morbidity Survey (NHMS), 2019 (Institute for Public Health (IPH), 2019). Interestingly, the NHMS found a lower prevalence of cigarette smoking among respondents with tertiary education (*n* = 2,426, 21.9%) compared to those with primary (*n* = 2,540, 23.0%) and secondary education (*n* = 5,442, 49.2%) levels, challenging the notion that educational level is a reliable indicator of smoking behaviour (Institute for Public Health (IPH), 2019; Mohd Yusoff et al., 2022). Consequently, while gender-related data may offer insights applicable to the broader population, caution is warranted in generalising findings based solely on educational demographics.

The majority of smokers in this study disclosed a habit of daily smoking, closely followed by those who smoked infrequently. This aligns with findings from the Global Adult Tobacco Survey (GATS) 2011, which indicated that a significant proportion of current tobacco users reported daily smoking (20.9%, 95% CI [19.0–22.9]) compared to occasional smoking (2.3%, 95% CI [1.7–3.0]) (Institute of Public Health (IPH), 2012a). Furthermore, the study revealed that most smokers had initiated smoking within the past 5 years, with only a tiny fraction having smoked for over a decade. This contradicts past studies by Rahman et al. (2022) and Williams et al. (2021), having said that most smokers (72.9% and 80.6%) had smoked for more than 10 years. Additionally, the data indicated that the majority of participants smoked fewer than ten cigarettes per day, while a minority smoked more heavily, which was more than 20 cigarettes daily. This suggests a prevalence of light smoking among respondents, consistent with GATS 2011, which similarly identified a low proportion of heavy smokers exceeding 25 cigarettes per day (8.1%) (Institute of Public Health (IPH), 2012b).

Overall, non-smokers exhibited a significantly higher level of interaction with the pictorial warnings on cigarette packaging (label processing), including reading, thinking about, and discussing them, compared to smokers. This finding is consistent with a study by Ratneswaran et al. (2016), which observed that non-smokers engaged in more discussions about pictorial warnings than smokers. These results suggest that non-smokers perceive pictorial warnings as more significant than smokers. This assertion is further supported by research conducted in Laos by Sychareun et al. (2015), which found that non-smokers were more inclined to rate pictorial warnings as very important than smokers (69.1% *vs*. 59.8%).

In this research, non-smokers exhibited a greater level of awareness regarding the health consequences of tobacco use compared to smokers. However, both groups displayed significant knowledge regarding the health effects depicted on cigarette packs through pictorial warnings. The highest percentages of awareness were observed regarding the association between tobacco smoking and lung cancer, indicating that a majority of respondents recognised this correlation. This aligns with findings from a study by Rahman et al. (2015) conducted in Sarawak, where respondents acknowledged smoking as a cause of lung cancer. This heightened awareness may be attributed to the widespread public health campaigns highlighting the link between smoking and lung cancer.

Additionally, mouth cancer emerged as another well-recognised health consequence of tobacco smoking. Other studies have similarly found pictorial warnings depicting lung, mouth, and neck cancer among the most recognisable health warnings (Hamzah, Saub & Marhazlinda, 2021). However, awareness regarding other health consequences, such as gangrene, was notably lower. This discrepancy could potentially be attributed to a lack of education regarding the medical connection between smoking and these specific health warnings, particularly in the case of pregnancy-related images and gangrene.

In this study, it is revealed that individuals who do not smoke demonstrate heightened emotional reactions toward pictorial warnings in comparison to smokers across three distinct metrics: fear, disgust, and avoidance of viewing the images. This aligns with research conducted by Gantiva et al. (2022), which indicated that pictorial warnings are more impactful for non-smokers than smokers. The findings suggest that smokers may develop desensitisation to pictorial warnings due to repeated exposure to cigarette packaging, potentially diminishing the effectiveness of such warnings over time, as smokers are accustomed to them. Ratneswaran et al. (2016) similarly observed that pictorial warnings are more efficacious for non-smokers, attributing this discrepancy to smokers’ existing awareness of the hazards associated with smoking. Additionally, a more significant proportion of non-smokers reported experiencing heightened feelings of ‘fear’ and ‘disgust’ compared to smokers (Ratneswaran et al., 2016). The habitual exposure of smokers to cigarettes and health warnings may attenuate the impact of pictorial warnings, indicating that smokers might also manifest emotions such as denial or dismissal in response to these warnings.

Our research indicates that a significant portion of smokers perceive pictorial warnings as influential in prompting them to contemplate quitting tobacco usage. Specifically, our findings reveal that smokers frequently entertain thoughts of reducing cigarette consumption. This observation is consistent with past studies conducted in the United States, Spain, and France by Andrews et al. (2016), which underscored the efficacy of pictorial warnings in facilitating smoking cessation among smokers. Correspondingly, a survey conducted by Sychareun et al. (2015) found that most smokers expressed that pictorial health warnings were motivating factors to reconsider their tobacco habits. Furthermore, Fathelrahman et al. (2010) demonstrated that most smokers occasionally pondered the idea of quitting smoking upon encountering pictorial warnings and reflected on the associated health risks. Our study underscores the emotional impact of pictorial warnings, prompting contemplative thoughts such as considerations of quitting smoking, reducing cigarette consumption, and contemplating health risks. However, it is noteworthy that these warnings do not consistently deter smokers from continuing their smoking habit. Previous research by Pepper et al. (2020) suggests that some smokers may actively avoid looking at these warnings, potentially diminishing their immediate impact. Our findings suggest that many smokers are in a contemplation stage, acknowledging the issue but needing more readiness and confidence to enact behavioural changes regarding their smoking habits, thus not progressing beyond contemplation into action.

The impact of pictorial warnings on smoking initiation has predominantly been examined in non-smokers, focusing on their perceived impact on the inclination to smoke following exposure to such warnings. Previous research conducted by Auemaneekul et al. (2015) suggests that pictorial warnings play a significant role in dissuading non-smokers from initiating smoking. Auemaneekul et al. (2015) compared the effects of pictorial warnings and plain packaging, revealing a heightened intention not to smoke among non-smokers and former smokers after exposure to plain packaging compared to current smokers. Our findings align with this trend, indicating that a majority of non-smokers reported never experiencing an urge to smoke upon encountering pictorial warnings. This underscores the heightened awareness among non-smokers regarding the health risks associated with smoking, as facilitated by their comprehension of the dangers and consequences depicted in the warnings. Consequently, non-smokers may exhibit reduced susceptibility to the temptation of smoking, as the graphic imagery fosters a negative association with tobacco use, thereby diminishing its appeal among this demographic.

Our investigation into the most impactful images on cigarette packs reveals disparate responses from smokers and non-smokers towards pictorial warnings. Smokers identified lung cancer as the most impactful image, while non-smokers cited mouth cancer. This finding resonates with prior research by Hamzah, Saub & Marhazlinda (2021), indicating that pictorial health warnings concerning lung, neck, and oral health are most comprehensible and accepted by respondents. Additionally, Gantiva et al. (2022), found that images depicting cancers and abortion evoke more excellent emotional responses compared to others. Interestingly, our study found that respondents deemed gangrene as the least impactful image, suggesting a lack of awareness regarding this medical condition associated with smoking-related health risks. Hamzah, Saub & Marhazlinda (2021) emphasise the importance of crafting comprehensible messages in pictorial warnings, particularly avoiding medical terminology such as ‘gangrene’ and focusing on easily understood language accessible to individuals with diverse educational backgrounds and cultural perspectives. Therefore, providing explanatory notes alongside health warning labels could enhance understanding. Our findings underscore the necessity of integrating insights from health professionals and considering the knowledge levels of target audiences during the development of pictorial warning labels.

From a policy perspective, these findings highlight the need to evaluate and enhance continuously. Although pictorial warnings have proven effective in raising awareness, especially among non-smokers, their limited impact on smoking cessation among smokers suggests that additional interventions are necessary. Policymakers should consider adopting more aggressive tobacco control strategies, such as increasing the size of pictorial warnings on cigarette packaging or implementing plain packaging, which has been shown to reduce the appeal of tobacco products, especially among young adults and new smokers. Public health campaigns targeting specific demographics, such as males and lower-educated populations, should be intensified to close the gaps in awareness and smoking behaviour. Furthermore, policies encouraging frequent rotation of warning images and incorporating culturally relevant messages could enhance their effectiveness.

Future research should explore more personalised and dynamic approaches to messaging. Studies could investigate whether rotating more graphic and emotionally charged images could mitigate the desensitisation observed among smokers over time. In addition, research should assess the potential benefits of combining pictorial warnings with other cessation aids, such as counselling services or digital interventions, which could help move smokers from contemplation to action. Longitudinal studies tracking smoking behaviour over extended periods would also provide valuable insights into the long-term effects of pictorial warnings on smoking cessation, particularly in light of changing trends in tobacco use and public health messaging.

Finally, future investigations should explore the psychological mechanisms behind why specific images (such as lung or mouth cancer) resonate more with different groups. Understanding these emotional triggers can inform the design of more targeted and impactful warnings. Researchers should also look into the role of socioeconomic and cultural factors in shaping responses to pictorial warnings, ensuring that future tobacco control policies are inclusive and resonate with diverse population groups.

By addressing these areas through policy enhancements and targeted research, there is potential to significantly strengthen the effectiveness of pictorial warnings in both preventing smoking initiation and promoting smoking cessation.

### Limitations

The key limitation of this study is that the utilisation of a cross-sectional structured survey presents another challenge, as respondents may be inclined to provide socially desirable responses, particularly regarding sensitive topics like smoking. This bias could lead to underreporting of smoking or overreporting of intentions to quit among smokers. At the same time, non-smokers may hesitate to admit any inclination towards starting smoking due to fear of judgment.

## Conclusions

This study highlights the differing perceptions and impacts of pictorial warnings on cigarette packaging between smokers and non-smokers in Malaysia. While non-smokers demonstrated greater engagement with and emotional responses to the warnings, smokers showed less frequent interaction and a tendency toward desensitisation. Although pictorial warnings play a vital role in raising awareness of the health risks of smoking, particularly lung and mouth cancer, their effectiveness in encouraging smoking cessation among smokers remains limited. The findings suggest that additional, more aggressive tobacco control policies, such as more significant warnings or plain packaging, may be needed to reduce smoking rates further.

Future research should focus on refining the design and messaging of pictorial warnings, possibly incorporating more graphic or culturally relevant images to increase their impact. Longitudinal studies that track behaviour over time, combined with complementary cessation aids, could offer more insight into how best to move smokers from contemplation to action. Including socioeconomic and psychological factors in future studies would provide a more comprehensive understanding of how to tailor warnings to diverse audiences. By addressing these areas through targeted policies and research, there is significant potential to enhance the overall effectiveness of tobacco control strategies in reducing smoking prevalence and preventing smoking initiation.

In conclusion, smokers generally perceived pictorial warnings on cigarette packaging as less significant and showed limited engagement with the warning labels compared to non-smokers. On the other hand, non-smokers exhibited a greater understanding and interpretation of these warnings. While the implemented pictorial warnings may serve as an initial step towards encouraging smoking cessation, they have limited effectiveness in deterring smokers from continuing the smoking habit. Conversely, non-smokers demonstrated a slight increase in knowledge and awareness regarding the health consequences of tobacco use as compared to non-smokers. This study underscores the gap in awareness and offers valuable insights for policymakers in designing future smoking cessation campaigns, aligning with the World Health Organization’s efforts. MPOWER policies include monitoring tobacco consumption and the effectiveness of preventive measures, protecting people from tobacco smoke, offering help to quit tobacco use, warning about the dangers of tobacco, enforcing bans on tobacco advertising, promotion and sponsorship, and raising taxes on tobacco to combat tobacco usage globally. Hence, future studies should focus on re-evaluating other suitable pictorials on cigarette packaging.

## Supplemental Information

10.7717/peerj.18713/supp-1 Supplemental Information 1 Final Overall data (English).

## Additional Information and Declarations

### Data Availability

The following information was supplied regarding data availability: The raw data is available in the Supplemental File.
